# Generalized Bayesian method for diagnostic classification models

**DOI:** 10.1017/psy.2024.20

**Published:** 2025-01-03

**Authors:** Kazuhiro Yamaguchi, Yanlong Liu, Gongjun Xu

**Affiliations:** 1 University of Tsukuba, Tsukuba, Japan; 2 University of Michigan, Ann Arbor, MI, USA

**Keywords:** diagnostic classification models, generalized Bayesian method, loss function-based method, parameter estimation

## Abstract

This study extends the loss function-based parameter estimation method for diagnostic classification models proposed by Ma, de la Torre, et al. (2023, Psychometrika) to consider prior knowledge and uncertainty of sampling. To this end, we integrate the loss function-based estimation method with the generalized Bayesian method. We establish the consistency of attribute mastery patterns of the proposed generalized Bayesian method. The proposed generalized Bayesian method is compared in a simulation study and found to be superior to the previous nonparametric diagnostic classification method—a special case of the loss function-based method. Moreover, the proposed method is applied to real data and compared with previous parametric and nonparametric estimation methods. Finally, practical guidelines for the proposed method and future research directions are discussed.

## 1 Introduction

Learning is an important aspect of human life. The current status of individual knowledge or depth of understanding must be evaluated to ensure efficient learning. Test analysis models called diagnostic classification models (DCMs; Rupp et al., 2010; von Davier & Lee, 2019) have been popularly employed to capture an individual’s learning status. Notably, DCMs provide useful statistical tools to reveal individuals’ current learning status based on the test’s item responses. Latent knowledge or cognitive elements are called attributes and are expressed as latent categorical variables in DCMs. Moreover, DCMs are known as restricted latent class models (e.g., Rupp & Templin, 2008; Xu, 2017), wherein each possible set of attributes represents a latent class. In other words, attribute mastery patterns indicate the attributes that are either mastered or not mastered. Therefore, one of the DCMs’ final outputs is the estimate of the attribute mastery patterns of individuals or attribute mastery probabilities.

Various parameter estimation methods for the DCMs have been actively developed. Parametric and nonparametric estimation methods are commonly used in DCMs. Parametric estimation methods assume parametric item response functions and structural models. Therefore, parametric estimation methods employ a likelihood function under the assumed model and include (penalized or regularized) maximum likelihood estimation (e.g., Chen et al., 2015; de la Torre, 2009; Ma, Ouyang, & Xu, 2023) and Bayesian estimation methods (e.g., Culpepper, 2015; Yamaguchi & Okada, 2020; Yamaguchi & Templin, 2022b), incorporating prior distributions for model parameters. Numerous parametric estimation methods have been developed and their properties have been studied (e.g., von Davier & Lee, 2019).

On the other hand, nonparametric methods (e.g., Chiu et al., 2018; Chiu & Douglas, 2013) do not use probabilistic item response models; instead, they use an ideal response to define a type of discrepancy function, which will be formally defined in a later section. Such discrepancy functions are defined based on the distance between each item’s ideal and actual responses. Intuitively, nonparametric methods directly estimate attribute mastery patterns, which minimize the discrepancy function. Therefore, nonparametric methods do not require a probabilistic item response function. Nonparametric methods exhibit satisfactory statistical properties, such as consistency under certain conditions (Chiu & Köhn, 2019; Wang & Douglas, 2015).

Recently, a general parameter estimation method that can uniformly express parametric and nonparametric methods was developed by Ma, de la Torre, and Xu (2023). The unified estimation method developed by Ma, de la Torre, and Xu (2023) is a loss function-based estimation method for DCMs. If we select cross-entropy for a loss function, its minimization corresponds to maximizing the joint maximum likelihood (Chiu et al., 2016). The distance or discrepancy function in nonparametric methods is a well-known loss function. Additionally, by adding penalty terms to a cross-entropy loss function, we obtain the maximum a posteriori (MAP) estimates, classical Bayesian estimates, to minimize it. These examples indicate that the loss function-based estimation method is flexible and can represent various estimation methods in a unified manner. Furthermore, a unified estimation algorithm for the loss function-based estimation method was available.

However, loss function-based methods exhibit certain limitations. First, these methods only provide point estimates, which may be problematic because we cannot evaluate how point estimates vary due to sampling or estimation variations. Therefore, we cannot evaluate the uncertainty of attribute mastery using the loss function-based method. Furthermore, attribute mastery probabilities for each individual are not expressed in the loss function-based method. This is the same problem that occurs in DCMs’ nonparametric estimation method. However, attribute mastery probabilities represent a more nuanced situation than attribute mastery pattern results, with or without mastery. Another limitation of these methods is that prior information on weight parameters in the generalized nonparametric method that defines generalized ideal responses is generally not considered. However, DCM users may have prior knowledge of the test items’ conjunctive and disjunctive nature. If so, domain-specific knowledge must be included to improve parameter estimates.

It is not only loss function-based methods that have limitations that need to be addressed; several limitations of previous parametric and nonparametric estimation methods likewise need to be noted. First, parametric estimation methods need to specify data-generating distributions, which determine the likelihood function. The likelihood function provides a connection between data and model parameters such as attribute mastery patterns. Moreover, likelihood functions make it possible to evaluate estimation uncertainty with the asymptotical theory within the maximum likelihood framework or the posterior distribution within the Bayesian framework. However, the data-generating process is not always specified. DCMs are part of the educational measurement model family that need various constraints and limitations, making it difficult to specify the model.

Some of the limitations of the nonparametric methods are the same as those of the loss function-based methods. For instance, current nonparametric methods for DCMs cannot evaluate the uncertainty of attribute mastery estimates. The nonparametric methods for DCMs were developed in studies with small sample sizes (Chiu & Douglas, 2013). Ultimately, the nonparametric method for DCMs can be applied to individuals; however, the parameter estimates need to be evaluated with variability of parameter estimates. Currently, the nonparametric methods simply select attribute mastery patterns to minimize prespecified distance functions so the parameter uncertainty evaluation is not included in the framework. The parameter estimates with nonparametric methods can be changed by small differences in the loss function. One main purpose of DCMs is the diagnosis of individual knowledge. Thus, such variations in parameter estimates due to small differences in the distance functions may be a fundamental problem for application.

To overcome these limitations and extend the previous loss function-based estimation method for DCMs, we employ the generalized Bayesian (GB; Bissiri et al., 2016) method. The usual Bayesian parameter update determines the likelihood function and updates the model parameters in the likelihood with the observed dataset. By contrast, the GB method can express parameter updating with a dataset via loss functions. Therefore, Bayesian inference is applicable to nonparametric-based estimation methods as well as to likelihood-based methods. Moreover, other benefits of the GB method are as follows. First, the GB method originally assumes the 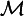 -open setting (Bernardo & Smith, 2009, Chap. 6), which implies that the GB method provides a valid inference even if the assumed model does not match the true data-generating mechanism. Various DCMs have been developed; however, selecting an appropriate item response function that expresses the true data-generating mechanism is not always possible. The GB method does not require an entire data-generating model but instead sets a loss function related to the parameter sets of interest. This means that we do not need to find a correct data-generating model, which is always unknown and often misspecified. We expect the GB method to overcome the practical difficulties of DCMs’ applications.

Second, the GB method allows the use of flexible loss functions and priors. The uncertainty of the parameters expressed in the loss functions is easily demonstrated in the generalized posteriors generated using the GB method. In other words, the GB method can handle the amount of uncertainty of attribute mastery estimates. Not only point estimates but also uncertainty variation are important for careful decisions in diagnostic evaluation. The GB method provides a useful tool for addressing the above problems, which both parametric and nonparametric methods have. Furthermore, the generalized posterior is easily obtained using a Markov chain Monte Carlo (MCMC) routine, such as the Metropolis–Hastings method. Third, we can control the relative importance between the dataset and the prior via the learning rate parameter. If the obtained data’s quality is questionable, an inference that is completely dependent on the data may lead to inappropriate decisions. In such cases, the data’s relative importance can be reduced. The learning rate parameter enables a more flexible inference.

Based on these discussions, we develop a GB method to overcome the limitations of the loss function-based estimation method for DCMs (Ma, de la Torre, & Xu, 2023). The remainder of this article is organized as follows: The second section demonstrates the basic setup of the DCMs and the previous loss function-based estimation method. The third section provides the GB method’s fundamentals and its application to DCMs based on their loss functions. Therein, the MCMC algorithm for a generalized posterior is also discussed. The GB method’s mathematical properties under certain conditions are discussed in the fourth section. The fifth and sixth sections comprise simulation and real data analysis examples of GB inference for DCMs, wherein we compare previous nonparametric estimation methods in a simulation study. Finally, the seventh section serves as the discussion, where the limitations of the GB inference and future directions of DCMs’ estimation methods are discussed.

## 2 Model setup and previous estimation methods

### 2.1 Model setup of DCMs

First, we express an individual’s attribute mastery pattern using a vector of length 
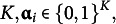
 where 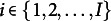 . The 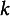 th element of the attribute mastery pattern vector 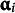 is 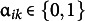 , where 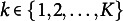 ; it takes one if individual 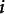 masters attribute 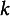 , and otherwise, it takes 0. In this study, we assume unconditional attribute mastery patterns, where all possible attribute mastery patterns and the number is 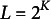 . Therefore, the 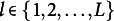 -th attribute mastery pattern can be written as 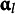 . The set of attribute mastery patterns for all individuals is 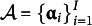 . To define the parametric measurement model, we also need to specify the diagnostic relationship between the attributes and item sets.

The diagnostic relationship between the attributes and test items is called the 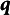 -vector, 
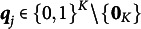
 , where 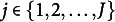 ; if the 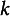 th attribute is required for item 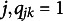 ; otherwise 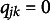 . Additionally, 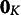 is a vector of length 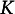 and all its elements are 0. Here, we assume there is no item with 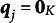 . The Q-matrix (Tatsuoka, 1985) is a 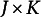 matrix defined by 
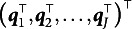
 .

Parametric DCMs define their measurement models using attribute mastery patterns and a 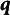 -vector. For example, one of the most general DCMs, known as the log linear cognitive diagnostic model (LCDM; Henson et al., 2009), uses an item parameter vector 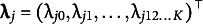 , and the measurement model of 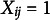 , which is a conditional response probability of individual 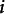 for item 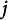 , is:
(1)

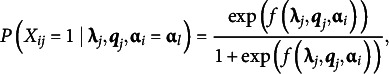

where 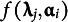 is:
(2)

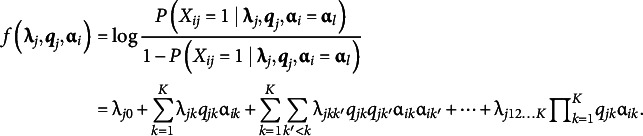



The LCDM has several parameters. The first parameter is the intercept 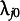 , which determines the baseline correct item response probability. Attribute mastery patterns that do not master any attributes requiring item 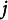 take response probability. The main effect parameters were 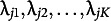 ; each parameter affected the correct item response probabilities with the corresponding attributes. The first-order interaction parameter 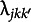 is the effect of simultaneously mastering the attributes 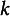 and 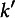 . Similarly, we introduce the highest interaction term as 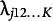 . General cognitive diagnosis models that are similar to LCDM have also been proposed in the literature, including the generalized DINA (GDINA) model (de la Torre, 2011) and general diagnostic model (GDM; von Davier, 2008).

As some attributes are not measured by item 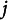 , the number of estimated item parameters under LCDM is 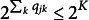 . Moreover, notably, one-to-one mapping exists between the LCDM item parameters and conditional item response probabilities (Rupp et al., 2010). Therefore, it is convenient to use conditional item response probabilities to develop parameter estimation methods. The same strategy was adopted in previous studies (Yamaguchi & Okada, 2020; Yamaguchi & Templin, 2022b), where DCMs are a restricted version of latent class models (e.g., Rupp & Templin, 2008; Xu & Shang, 2018).

Therefore, let the correct item response probability be parameter 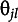 :
(3)

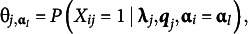



Additionally, the attribute mastery mixing parameters 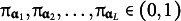 are defined as 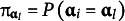 , satisfying 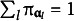 . From this notation, the complete data likelihood function of the LCDM is:
(4)

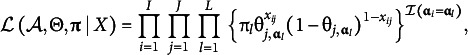

where 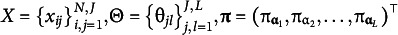 , and 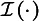 is an indicator function.

We add some remarks on the correct item response probabilities for item 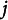 . First, as mentioned previously, some attribute mastery patterns have the same item response probabilities because of the setting of the 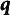 vector. Moreover, some submodels of the LCDM assume fewer parameters than the general LCDM and have parsimonious model forms. The model settings for each item can differ, but we assume that all test items have the same general LCDM form.

Second, the correct item response probabilities for item 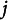 exhibit an ordinal relationship: These relationships are known as monotonicity constraints (Xu & Shang, 2018). The formal expression of the monotonicity constraints proposed by Xu and Shang (2018) is:
(5)

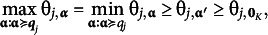

where we write 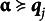 if 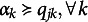 ; otherwise, 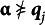. These constraints imply that the patterns mastering all skills measured in item 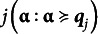 should have the highest of all the patterns. By contrast, all nonmastering patterns had the lowest correct item response probability. The middle mastering patterns satisfying 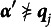 have response probabilities between these two probabilities.

### 2.2 Loss function-based parameter estimation

This section introduces the loss function-based parameter estimation method proposed in Ma, de la Torre, and Xu (2023). First, we describe certain elements of the loss function-based method. In this framework, we introduce the length 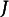 centroid parameter vector: 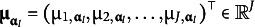 . Additionally, a penalty term for the mixing parameter 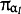 is introduced as 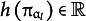 . Furthermore, an element-wise loss function taking item response vector 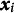 and a centroid parameter vector 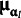 is expressed as 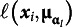 ; its codomain is real positive number 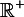 . The 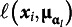 is the individual-level loss function. Therefore, the loss function of the entire dataset is based on the individual-level loss function:
(6)

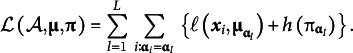



The second summation takes over the individuals with the attribute mastery pattern 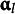 .

Parameter estimates are obtained to minimize the loss function defined above:
(7)

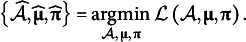



Directly minimizing the above loss function is not easy; therefore, we use the iterative update rule instead. In the estimation algorithm, we first set initial parameters 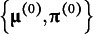 . When we have parameter estimates at 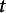 th iteration, 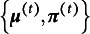 , the following update steps are repeated:
(8)

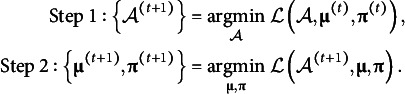



If the predetermined convergence criterion is satisfied, for example, 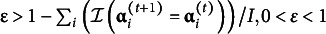 , the update process is stopped, and the parameter estimates become output: 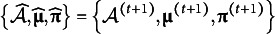 .

Many previous estimation methods can be viewed as special cases of the general loss function formulation framework. The joint likelihood estimation of the parametric DCM is a first example. In the following, we focus on the deterministic inputs noisy, “and” gate model (DINA model; Junker & Sijtsma, 2001; MacReady & Dayton, 1977; Maris, 1999) as an example because it is well-known and considered the most parsimonious DCM. The loss function further used to obtain the MAP estimation, which is the negative of the sum of the log-likelihood and log-prior density functions, is also presented. Subsequently, a nonparametric classification method (NPC; Chiu & Douglas, 2013; Wang & Douglas, 2015) and generalized NPC (GNPC; Chiu & Köhn, 2019; Chiu et al., 2018) are formulated under the above framework. Furthermore, NPC and GNPC are extended to the GB framework in a later section.

The DINA model is the simplest and most fundamental DCM, which is a special case of the LCDM. The DINA model assumes only the intercept and the highest interaction terms of the LCDM item parameters. Let the subscript set of attributes measured by item 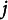 be 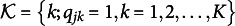 and let the LCDM kernel for the DINA model be reduced to:
(9)

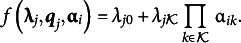



In the conventional DINA formulation, two-item response probabilities are represented by estimating the 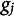 and slipping 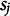 parameters:
(10)

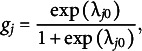



(11)

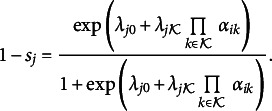



The guessing parameter 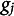 indicates the chance level of a correct item response for attribute mastery patterns that lack at least one attribute required by item 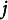 . The slipping parameter 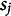 is the incorrect response probability of all-mastering-attribute mastery patterns required by item 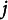 . Both 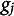 and 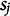 can be represented as functions of the ideal responses
(12)

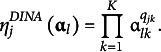



The ideal response represents the response of an individual who belongs to the 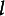 th attribute mastery pattern for item 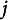 without errors. Then, 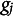 and 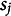 are represented as conditional probabilities:
(13)

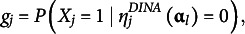



(14)

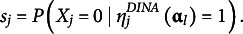



Using the item response probabilities, the centroid parameter under the DINA model is:
(15)

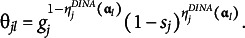



Assuming a cross-entropy loss, which is 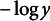 , the likelihood-based loss function for the DINA model is:
(16)

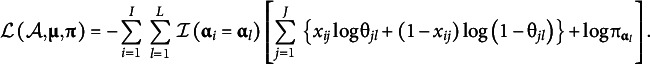



We assume 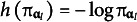 . The loss function defined in Equation 16 is equivalent to a negative log complete likelihood function. Therefore, minimizing equation 16 corresponds to maximizing the likelihood function; the minimizers 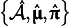 can be considered as the maximum likelihood estimate.

Subsequently, we examine the NPC and GNPC methods. Following Chiu and Douglas (2013), the loss function in the NPC method is defined by the Hamming distance between the individual item response vector and the ideal response vector:
(17)

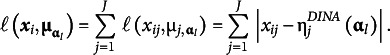



In the NPC method, the centroid parameter is the ideal response 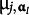 . The NPC estimates are obtained to minimize Equation 17 for each individual:
(18)

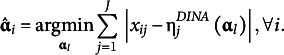



Clearly, the Hamming distance is a loss function, and the NPC method is a loss function-based estimation method.

As introduced in Chiu et al. (2018), the GNPC is a type of generalization that employs DINA-type and deterministic inputs noisy, “or” gate (DINO; Templin & Henson, 2006)-type ideal responses to define a generalized ideal response. The DINO-type ideal response is
(19)

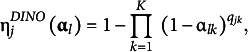

and 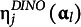 becomes one if pattern 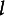 masters at least one attribute required for item 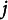 ; otherwise, it becomes 0. The generalized ideal response is then defined as
(20)

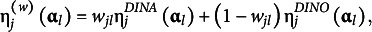

where 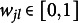 is a weight parameter that determines an item’s tendency. If the item is more like DINA or conjunctive, 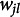 is close to one. By contrast, a 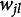 near zero means that the item is DINO-like or disjunctive in nature. The GNPC assumes a Euclidean distance for its loss function
(21)

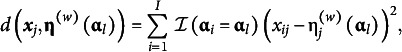



where 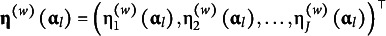 . The weight parameter is estimated via 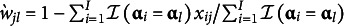 . The loss function of the GNPC is
(22)

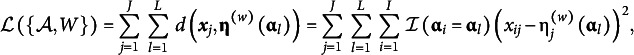



where 
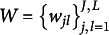
 . The GNPC requires iterative updates of weight 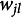 and attribute mastery patterns. The detailed update rule is described in Chiu et al. (2018). Note that if 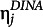 and 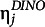 are not distinguished for some items and attribute patterns, the weight value is fixed to a value close to zero or one. See Chiu et al. (2018) for a detailed discussion.

As demonstrated above, parametric and nonparametric estimation methods can be treated in a unified loss function-based framework (C. Ma, de la Torre, & Xu, 2023). However, these loss function-based parameter estimates usually only provide point estimates, and uncertainty quantification in the parameter estimates has been considered less serious. Furthermore, different specifications of the measurement model precipitate significantly different attribute mastery patterns (e.g., Li et al., 2016). However, assessing all possible measurement models for all test items may be difficult. The GNPC is a promising estimation method that can be used in varied situations, even when the measurement model is unknown. However, prior knowledge of the weight parameters in the GNPC is often not considered. These problems can be solved using the GB method introduced in the following section.

## 3 GB method for DCMs

### 3.1 Construction of the generalized posterior

The GB method is a decision theory under a model misspecification situation (Bissiri et al., 2016). In other words, the assumed model may not accurately represent the true data-generating process, or the relationship between the model parameters and data may not be described via the assumed model, which is known as the 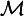 -open situation (Bissiri et al., 2016, p. 1111). The GB method is a coherent belief update procedure that uses a loss function even in the 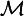 -open situation. Thus, the GB method extends the applicability of the typical Bayesian methods, which require a likelihood function.

Let datasets and parameter sets be 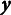 and 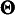 , respectively. Additionally, the loss function and prior distribution are 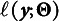 and 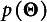 . Then, the generalized posterior of the parameter 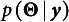 is:
(23)

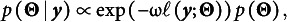

where 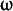 is called the learning rate, a tuning parameter that controls a dataset’s importance. Methods for determining the learning rate are still being studied (Wu & Martin, 2023); notably, no standard has been established thus far. The generalized posterior function is the result of updating the prior distribution based on the loss function. If we select a negative log-likelihood function for the loss function and 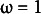 , the generalized posterior becomes the usual Bayesian posterior function.

Bissiri et al. (2016), pp. 1106–1107) discussed some of the validity requirements for loss functions. First, the solution of the loss function must exist. Second, the loss function must satisfy the following condition:
(24)

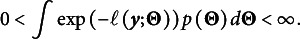



Some major loss functions considered in this study, such as the Hamming distance, Euclid distance, or cross-entropy loss, satisfy the above integral conditions. Additionally, Bissiri et al. (2016), p. 1107) identified natural assumptions for deriving a generalized posterior from a loss function. We should also point out that we only need to construct loss functions given a set of data for only the parameter of interest to employ the GB method. In this article, the GB method employed the loss function for attribute mastery patterns. The loss function is based on the GNPC: quadratic of Euclid distance. It satisfies the above conditions and is valid.

### 3.2 General form of the GB method for DCMs

The general form of the GB method for DCMs can be expressed using Equations 6 and 23:
(25)

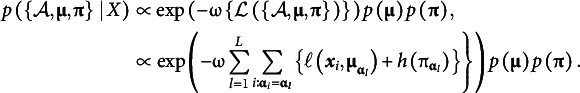



The penalty term was 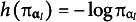 .

Using the GNPC loss function defined in Equation 22 and adding a penalty term for the mixing parameter 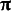 , the generalized posterior is:
(26)

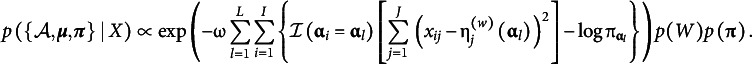



Notably, we treat weight 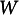 as a parameter and assume a prior instead of a centroid parameter 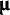 because the centroid parameter 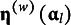 is determined by two ideal responses and weight parameters; thus, it is natural. Priors for the mixing parameters and weight parameters are assumed Dirichlet and Beta distributions:
(27)

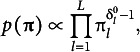



(28)

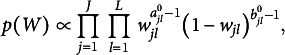



where 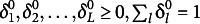 , and 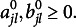


The posterior was numerically obtained using MCMC techniques, such as Metropolis–Hastings within the Gibbs sampling method, or MCMC software, such as JAGS (Plummer, 2003) or Stan (Carpenter et al., 2017). The conditional distribution of 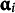 is categorical:
(29)

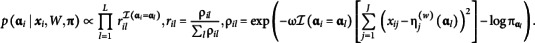



The conditional distribution of the mixing parameters was a Dirichlet distribution:
(30)

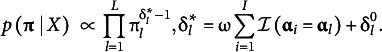



The conditional distribution of the weight parameter is not easily expressed; therefore, its MCMC update was performed using the Metropolis–Hastings method. The candidate was generated by a random walk using a uniform distribution: 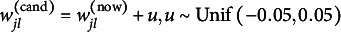 . Using the above distributions and updating rules, the MCMC for a generalized posterior is numerically approximated as follows: The mixing and weight parameters were initialized as 
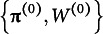
 , and the hyperparameters were set to 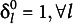 and 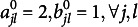 for example. Then, at the 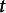 th MCMC iteration 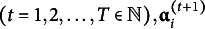 is generated from the categorical distribution expressed in Equation 29 with the 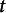 th MCMC sample of the parameter set at 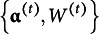 . The 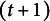 th MCMC sample of the mixing parameter is generated from the Dirichlet distribution shown in Equation 30 using 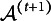 . 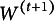 is obtained using the Metropolis–Hastings method.

Under the hyperparameter setting, the Dirichlet distribution for mixing parameters becomes a uniform distribution. This represents a scenario in which we have no information about the population attribute mastery ratio. In this means, the prior of the attribute mastery pattern has almost no information. The mean and SD of the prior of the weight parameter are 0.667 and 0.236, respectively. Under this setting, interval 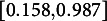 covers 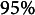 of the support of the parameter. The data analyst expected the items in the test to have a slightly conjunctive nature, which means the items behave more like in the DINA model than in the DINO model. However, the expectation is not particularly strong because the interval covering 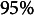 of the support of the parameter is wide. This interpretation indicates that the prior conveys some information about the weight parameters.

## 4 Mathematical properties of the proposed method: Consistency of the MAP estimators

First, we formally introduce the estimators under the GB framework and subsequently discuss their statistical behaviors under certain conditions. The appendix provides the full proofs. In this work, we assume that the item responses were generated from the Bernoulli distribution with parameter 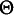 defined by Equation 3; the attribute mastery patterns were generated from a categorical distribution with a mixing parameter 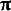 . Although several alternatives exist, MAP estimation provides a relatively natural and simple choice. Furthermore, MAP estimators of the GB method 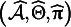 are estimators of the true parameters 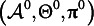 in the data-generating process. These are obtained by minimizing the loss function of 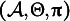 under the constraint imposed by the Q-matrix, as follows:
(31)

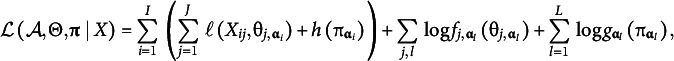

where 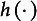 is a continuous nonincreasing regularization function of the proportion parameters 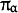 , often taken as 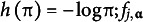 and 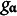 are the prior density functions of 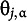 and 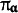 , respectively. Note that we consider a model sequence indexed by 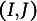 , where both 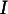 and 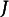 tend to infinity, while 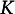 is held constant.

Several regularity conditions are required to ensure the consistency of MAP estimators. The first assumption is as follows.

Assumption 1. There exists 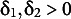 such that

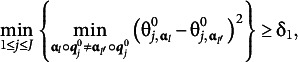


*and* 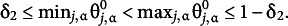


The first condition in Assumption 1 serves as an identification condition for local latent classes at each item level. The gap denoted by 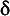 measures the separation between the latent classes, thereby quantifying the signals’ strength. The second condition in Assumption 1 keeps the true parameters away from the boundaries of the parameter space to prevent unusual behaviors of the element-wise loss.

Assumption 2 pertains to the discrete structures of 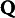 and is expressed as the following.

Assumption 2. All proportion parameters 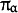 are strictly greater than zero, and there exist 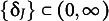 such that
(32)

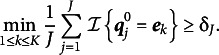

This assumption holds that 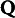 includes an increasing number of identity submatrices, 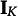 , as 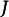 grows. Notably, by attaching the subscript 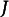 to the lower bound (32) in Assumption (2), we allow it to decrease to zero as 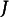 approaches infinity. As the following theorems show, if the rate at which variable 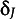 decreases meets certain mild requirements, the consistency of 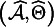 can be ensured.

The subsequent assumption concerns the element-wise loss function 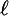 .

Assumption 3. The loss function 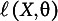 is twice continuously differentiable in 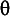 on 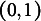 and 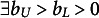 such that 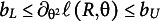 for 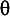 in a compact subset of 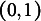 . The total loss (31) is minimized at class means given the subjects’ membership, as in, 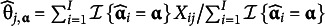 .

Assumption 3 imposes smoothness conditions on the element-wise loss function, rendering it convex. The upper bound of the second derivative is necessary to control the remaining term in the expansion of the first-order condition, and the lower bound allows us to quantify the estimator drift caused by the given priors. For the sample average assumption, we can verify that both 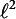 and cross-entropy loss functions satisfy Assumption 3.

Assumption 4 states that the true parameters minimize the element-wise loss functions and quantify the deviations when 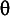 is not a true parameter. This assumption is expressed as follows:

Assumption 4. There exist constants 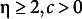 such that
(33)

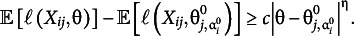

Assumption 4 holds for both the 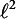 loss and the cross-entropy loss.

Assumption 5 is a technical assumption that allows us to control the effects of prior distributions on the estimators.

Assumption 5.

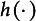
 in (31) is a continuous nonincreasing function of the proportion parameters, and 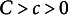 exists such that for any 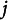 and 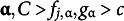 on a compact parameter subspace of 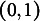 .

We can verify that the Dirichlet and Beta distributions satisfy this assumption.

Under the aforementioned regularity conditions, we demonstrate the consistency properties of the GB method with constraints for different attribute mastery patterns 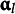 and 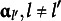 (Ma, de la Torre, & Xu, 2023; Xu, 2017):
(34)

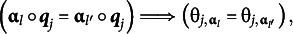

where 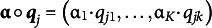 denotes the element-wise product of binary vectors 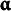 and 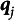 . This implies that the item response parameter 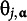 depends only on whether the attribute mastery pattern 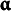 contains the required attributes 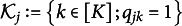 for item 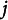 .

Based on the above five assumptions, we can derive consistent results for the GB method. The following main theorem first validates the clustering consistency of the GB method under the constraint (34), providing a bound for its convergence rate in recovering the attribute mastery patterns.

Theorem 1 (Clustering Consistency). Consider 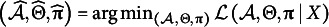 under the constraint (34). When 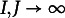 jointly, suppose 
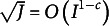
 for some small constant 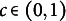 . Under Assumption 1 to Assumption 5. the clustering error rate is:
(35)

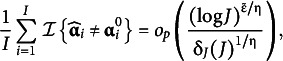

where for a small positive constant 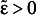 .

Theorem 1 bounds the error of the estimator 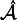 , which establishes the clustering consistency of the MAP estimators of the GB method, allowing the rate 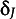 to go to zero. Notably, the scaling condition only assumes that 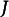 goes to infinity jointly with 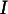 , but at a slower rate.

The following result demonstrates that the MAP estimator of the item parameters can be uniformly estimated consistently as 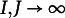 :

Theorem 2 (Item parameters consistency). Under Assumptions 1 to 5 and the scaling conditions given in Theorem 1, we have the following uniform consistency result for all 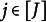 and 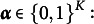



(36)

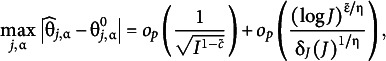

where 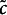 and 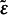 are small positive constants.

On the first error term, the condition 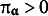 for all 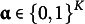 ensures that with probability one, there are enough samples within each class to provide accurate estimates of item parameters. Notably, 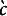 the first error term arises because the number of parameters approaches infinity jointly with the sample size 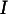 , which causes a slight deviation from the optimal error rate of 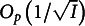 . The maximum deviation 
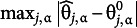
 is also affected by the classification error. This is indicated in the second error term 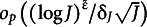 .

We can easily establish the consistency of the mixing parameter estimator 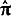 . When 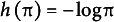 , the mixing parameters will be estimated as the sample average form 
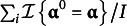
 , which converge in probability to 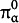 because of the clustering consistency.

Corollary 1 (Proportion parameters consistency). Under Assumptions 1 to 5 and the scaling conditions given in Theorem 1, when 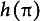 is taken as 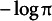 , we have 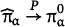 .

## 5 Simulation study

This section compares the previous (G)NPC and the corresponding GB methods using the loss functions in NPC and GNPC, named as GBNPC and GBGNPC, respectively. This simulation study primarily aims to assess the behavior of the GB method’s parameter estimates under finite small sample and item situations. As the GBNPC and GNPC are based on loss function in nonparametric methods, the most interesting parameters are attribute mastery patterns. In this simulation study, we mainly focus on the comparisons of the point estimates from these methods. To represent the uncertainty of the estimates, we also present attribute mastery probabilities using the GBGNPC and GBNPC methods, which indicate the benefit of the proposed method against the nonparametric methods.

The code for this simulation study is available on the Open Science Framework (OSF) webpage: https://osf.io/sau6j/.

### 5.1 Simulation settings

Five factors are manipulated in the simulations. All factors had two conditions; hence, 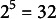 simulation settings were used. The first factor was the data-generating model: DINA or general DCM (e.g., LCDM). The DINA model condition is a simpler data-generating situation, whereas the general DCM model is more complex. The second factor was the Q-matrix; four or five attributes are listed in Tables 1 and 2. Table 1 contains 19 items: eight simple items (i.e., measuring only one attribute), six items measuring two attributes, five items requiring three attributes, and the most complex item measuring all four attributes. Table 2 lists 30 items: eight simple items, ten items measuring two attributes, and ten items measuring three attributes.

**Table 1:** The four-attribute 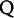 -matrix

Item	Attribute			
		1	2	3	4
1	1	0	0	0
2	0	1	0	0
3	0	0	1	0
4	0	0	0	1
5	1	0	0	0
6	0	1	0	0
7	0	0	1	0
8	0	0	0	1
9	1	1	0	0
10	1	0	1	0
11	1	0	0	1
12	0	1	1	0
13	0	1	0	1
14	0	0	1	1
15	1	1	1	0
16	1	1	0	1
17	1	0	1	1
18	0	1	1	1
19	1	1	1	1

**Table 2:** The five-attribute 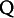 -matrix

Item	Attribute					Item	Attribute				
		1	2	3	4		5		1	2	3	4	5
1	1	0	0	0	0	16	0	1	0	1	0
2	0	1	0	0	0	17	0	1	0	0	1
3	0	0	1	0	0	18	0	0	1	1	0
4	0	0	0	1	0	19	0	0	1	0	1
5	0	0	0	0	1	20	0	0	0	1	1
6	1	0	0	0	0	21	1	1	1	0	0
7	0	1	0	0	0	22	1	1	0	1	0
8	0	0	1	0	0	23	1	1	0	0	1
9	0	0	0	1	0	24	1	0	1	1	0
10	0	0	0	0	1	25	1	0	1	0	1
11	1	1	0	0	0	26	1	0	0	1	1
12	1	0	1	0	0	27	0	1	1	1	0
13	1	0	0	1	0	28	0	1	1	0	1
14	1	0	0	0	1	29	0	1	0	1	1
15	0	1	1	0	0	30	0	0	1	1	1

Sample size was the third factor, with 30 or 300 participants assumed. The sample size setting of 30 participants mimicked classroom size. The sample size of 300 participants was 10 times larger than that of other classroom settings. The fourth condition was attribute correlation: independent 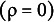 or highly correlated 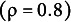 . The independent attribute condition was unrealistic but represented an ideal condition. The highly correlated condition was more realistic because the DCMs application indicated a high correlation among attributes (e.g., von Davier, 2008). The fifth condition was item quality. The high-item-quality condition indicates a high description of all nonmastering attributes and all perfectly mastering attributes. Under the high-item-quality condition, the correct response probability of the all nonmastering pattern was 0.1 and that of the all-mastering pattern was 0.9. On the other hand, in the low-item-quality condition, the corresponding probabilities were 0.3 and 0.7. The correct item response probabilities of the intermediate mastering patterns are generated based on Yamaguchi and Templin (2022b) or Yamaguchi and Templin (2022a).

The data generation process used herein was similar to those in previous studies, such as Chiu and Douglas (2013), Yamaguchi and Templin (2022b), and Yamaguchi and Templin (2022a). First, for each individual, we generated a continuous latent variable vector 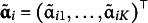 from 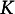 -dimensional normal distributions with zero means and compound symmetry covariance with a correlation of 0 or 0.8, and variances of 1. Subsequently, the continuous latent variable vector 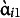 was converted into an attribute mastery pattern. More precisely, if 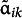 was greater than 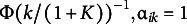 ; otherwise, 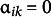 , where 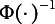 is the inverse cumulative normal distribution function. The simulated item responses were randomly generated using these attribute mastery patterns, an assumed data-generating model (DINA or general DCM), and item response probabilities. As mentioned in the previous section, the parameters of the priors in the GB method were set to 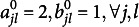 and 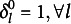 . The step size of the Metropolis update was fixed at 0.05. A one-chain MCMC with 1,000 iterations was employed. The first 500 iterations are discarded as the burn-in period; therefore, 500 MCMC samples were used to approximate the posterior distributions.

The main target parameter is attribute masteries, and they are categorical latent variables. However, common MCMC convergence criteria, such as Gelman-Rubin’s 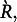 are for continuous variables, which means the indicators may not be applicable to categorical variables. Therefore, performing a convergence check of categorical variables in MCMC is not easy in this context. Instead of directly checking for the convergence of attribute mastery, we calculated the average correlations of the attribute mastery probabilities, which we estimated for the first and second halves of the MCMC iterations after the burn-in period. If the estimated results of the attribute mastery probabilities with the first half after the burn-in period are consistent with those of the later MCMC iterations, we consider the attribute mastery results to be stable.

The attribute mastery pattern of the 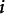 -individual was calculated based on the posterior attribute mastery probabilities. If the probability of the 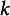 th attribute was <0.5, the attribute was considered mastered. Each estimation method was evaluated using two attribute mastery recovery indices: attribute-level agreement ratio (AAR) and pattern-level agreement ratio (PAR). AAR and PAR were calculated as follows:
(37)

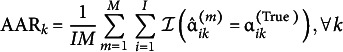



(38)

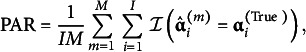



where 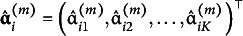 is an estimate of the attribute mastery pattern for individual 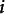 in the 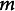 th simulation, and 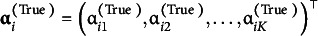 is the true attribute vector of individual 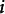 , where 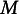 is the total number of simulations, which is 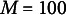 .

### 5.2 Results

Table 3 shows the results, which indicate correlations >0.98. Therefore, this result can be interpreted as an indication that the MCMC iterations were stable and attribute mastery can be estimated from the MCMC samples after the burn-in period.

**Table 3:** The average correlations of attribute mastery probabilities estimated by first and second halves of MCMC iterations after the burn-in period

Data generating	Sample	Attribute	Item	GBGNPC		GBNPC	
				model	size	correlation	quality				
								Three	Four	Three	Four
				attributes	attributes	attributes	attributes
DINA	30	0	High	.994	.994	.998	.998
						Low	.984	.983	.993	.994
				0.8	High	.996	.995	.998	.998
						Low	.984	.983	.995	.995
		300	0	High	.997	.998	.998	.999
						Low	.992	.993	.993	.995
				0.8	High	.999	.999	.999	.999
						Low	.992	.993	.995	.996
General	30	0	High	.990	.989	.996	.997
						Low	.981	.980	.994	.995
				0.8	High	.994	.994	.998	.998
						Low	.982	.982	.995	.995
		300	0	High	.997	.997	.997	.997
						Low	.992	.993	.994	.995
				0.8	High	.999	.999	.999	.999
						Low	.991	.993	.996	.996

*Note*: GBGNPC, generalized Bayesian method with generalized nonparametric loss function; GBNPC, generalized Bayesian method with nonparametric loss function.

**Figure 1:**
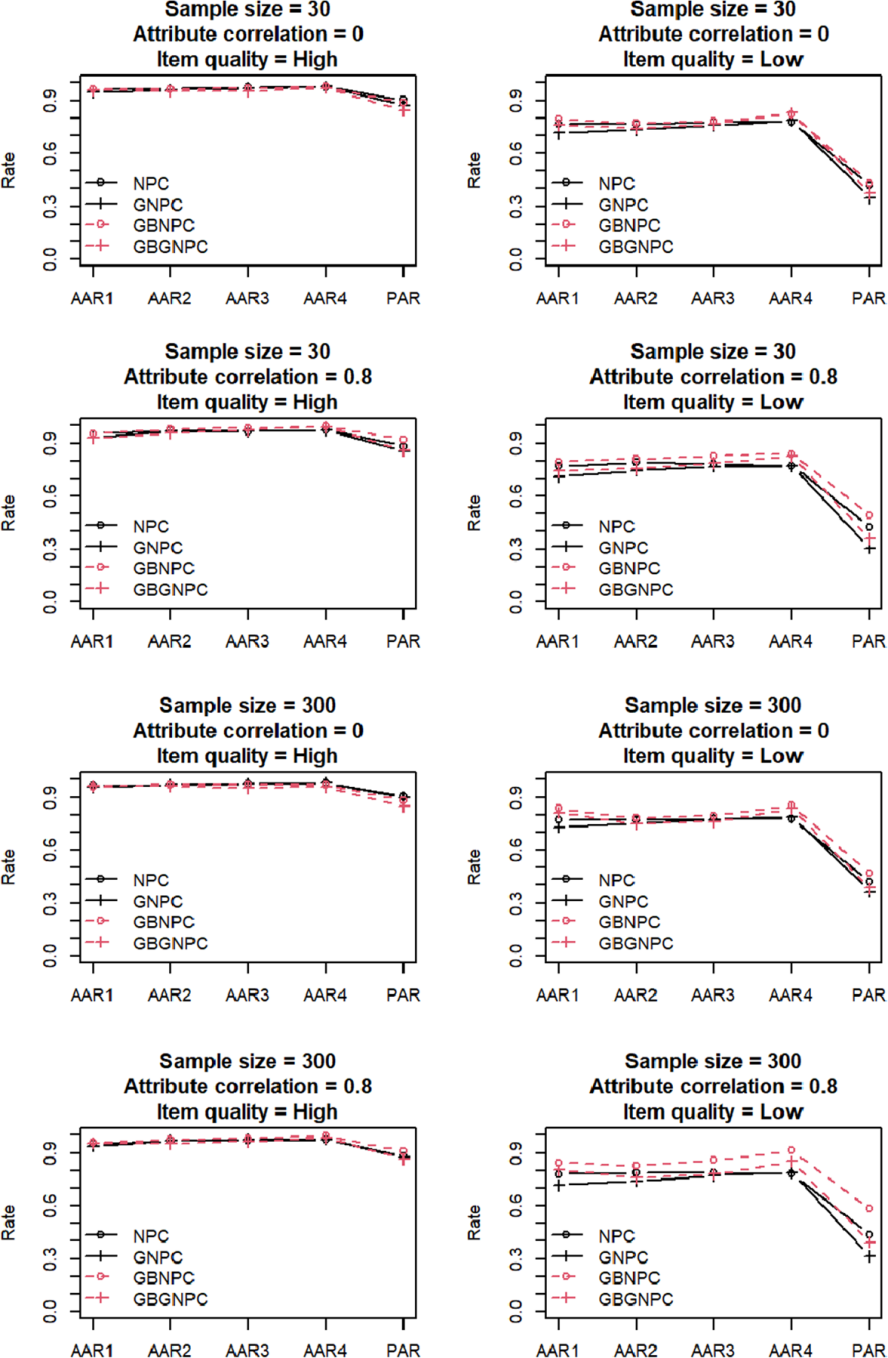
Simulation results of the DINA data generation with four-attribute 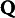 -matrix conditions.

**Figure 2:**
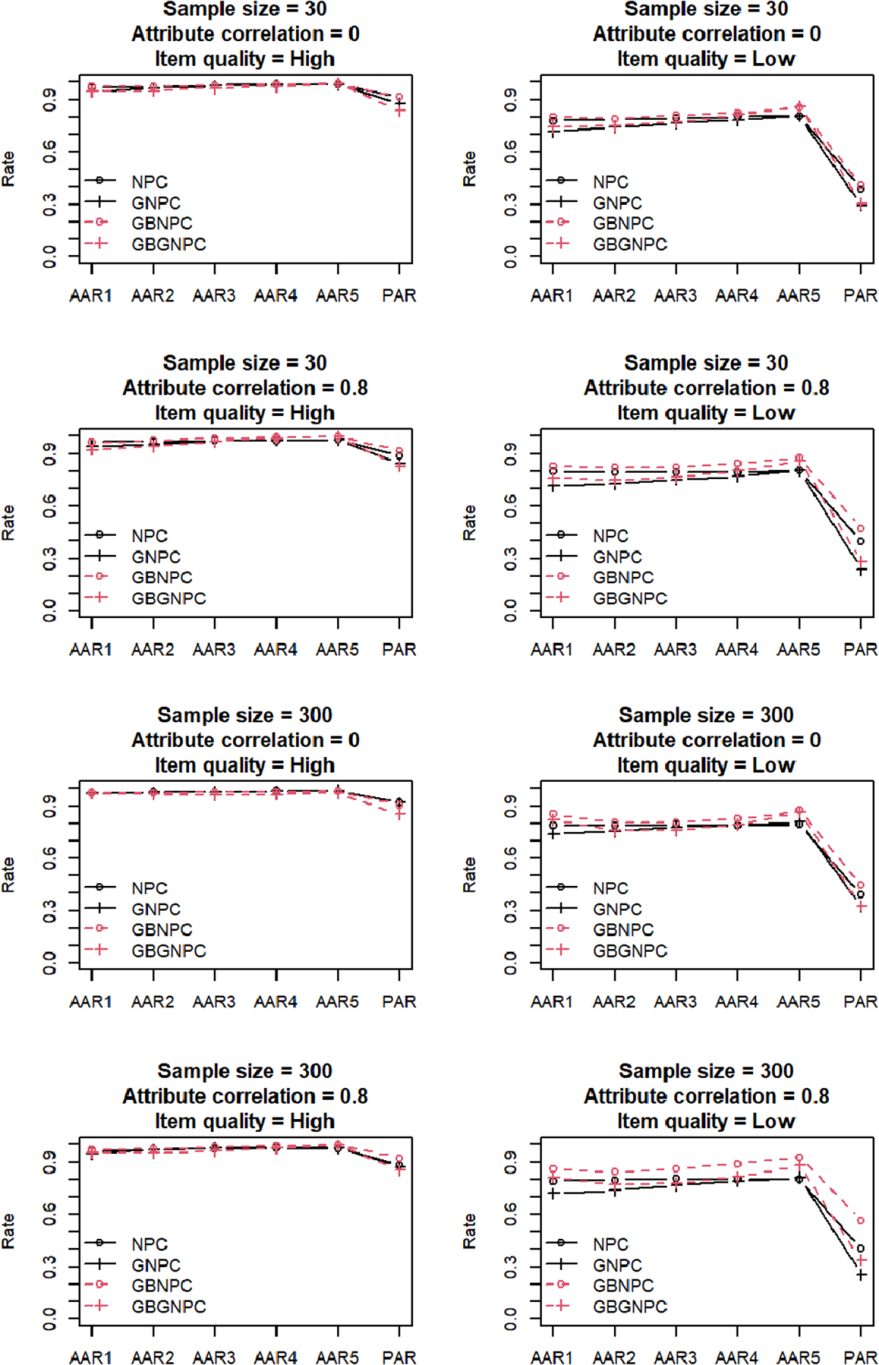
Simulation results of the DINA data generation with five-attribute 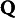 -matrix conditions.

Figures 1 and 2 present the simulation results of the DINA data generation with four- and five-attribute Q-matrix conditions, respectively. In this simulation, the AARs and PARs of the two Q-matrix conditions demonstrated similar tendencies; therefore, our discussion here focuses on the four-attribute Q-matrix condition. The high-item-quality conditions presented in the four left panels of Figure 1 indicated that all four estimation methods provide high AARs and PARs. The low-item-quality conditions presented in the four right panels of Figure 1 indicated lower AARs and PARs than the high-item-quality conditions, and the low-item-quality conditions exhibited some differences among the four estimation methods. Attribute correlations under low-item-quality conditions affected AARs and PARs more significantly. Furthermore, GBNPC and GBGNPC demonstrated higher AARs and PARs than the corresponding NPC and GNPC methods under the 30-sample size, 0.8 attribute correlation, and low-item-quality conditions. Interestingly, GBNPC exhibited the highest AARs and PARs under the 300-sample size, 0.8 attribute correlation, and low-item-quality conditions. Moreover, under these conditions, GBGNPC had similar AARs and PARs to the NPC, and the GNPC produced the least optimal result.

**Figure 3:**
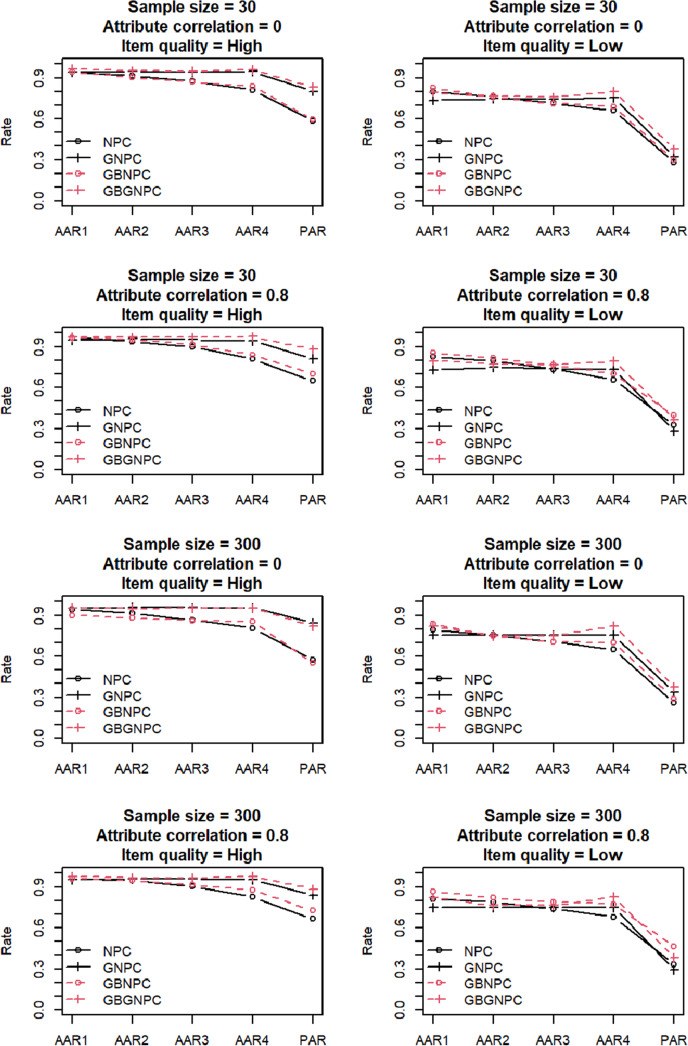
Simulation results of the general DCM data generation with four-attribute Q-matrix conditions.

**Figure 4:**
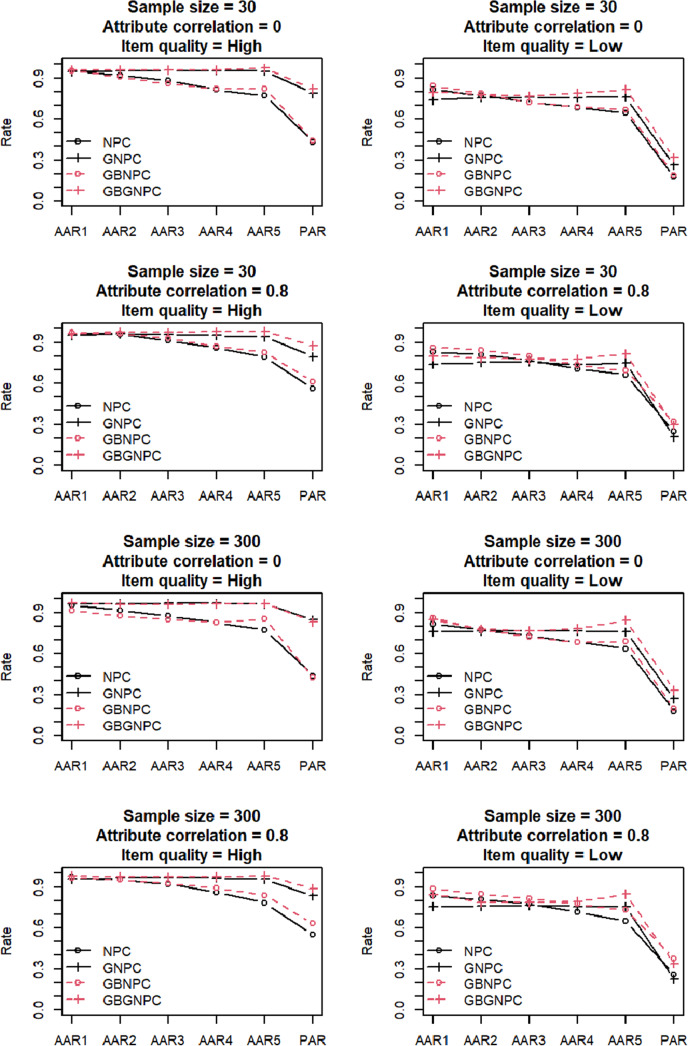
Simulation results of the general DCM data generation with five-attribute Q-matrix conditions.

Figures 3 and 4 present the results of the general DCM data generation with four- and five-attribute Q-matrix conditions, respectively. Again, the AARs and PARs of the two Q-matrix conditions exhibited similar patterns; hereafter, we predominantly focus on results of the four-attribute Q-matrix conditions. Under high-item-quality conditions, GNPC and GBGNPC outperformed NPC and GBNPC. Furthermore, under high-attribute correlation conditions, GBGNPC was superior to GNPC; the same pattern was observed between GBNPC and NPC under the same conditions. In the low-item-quality conditions presented in the four right panels of Figure 3, GBGNPC and GBNPC tended to have higher AARs and PARs than GNPC and NPC. In particular, a sample size of 300, a high-attribute correlation, and low-item-quality conditions indicated better AARs and PARs for GBGNPC and GBNPC than GNPC or NPC.

**Figure 5:**
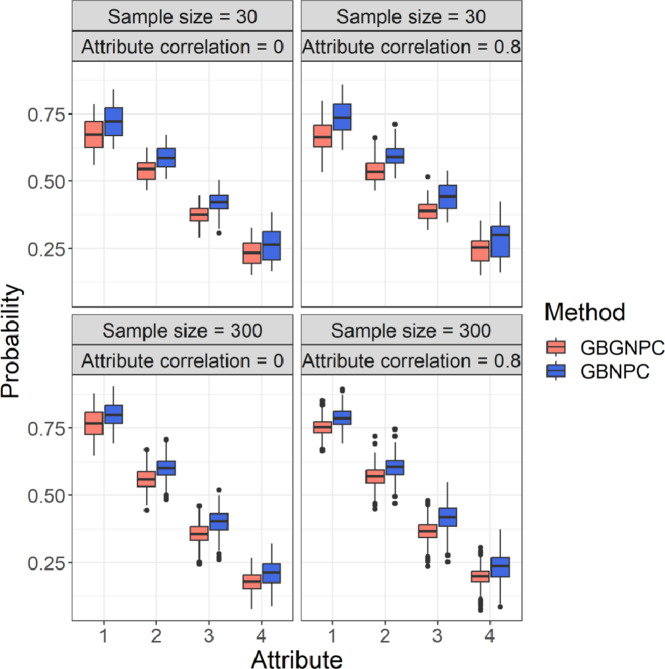
Box plots of attribute mastery probabilities of the DINA data generation with four-attribute Q-matrix conditions.

**Figure 6:**
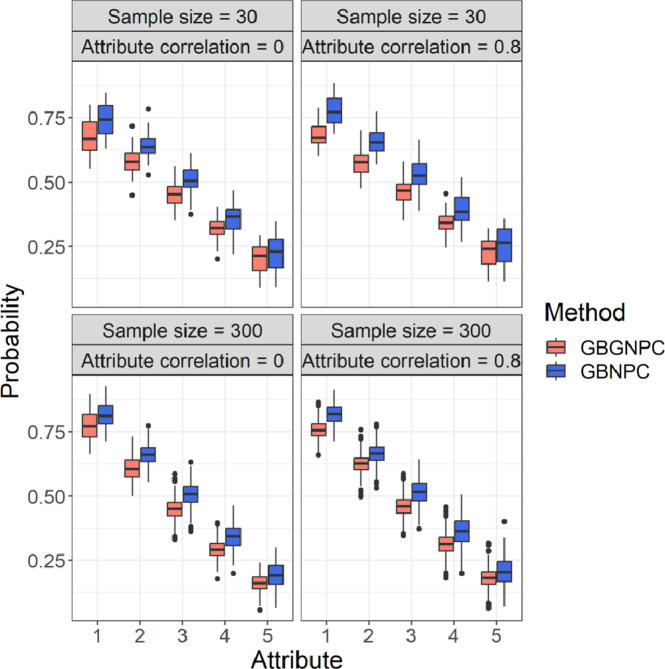
Box plots of attribute mastery probabilities of the DINA data generation with five-attribute Q-matrix conditions.

**Figure 7:**
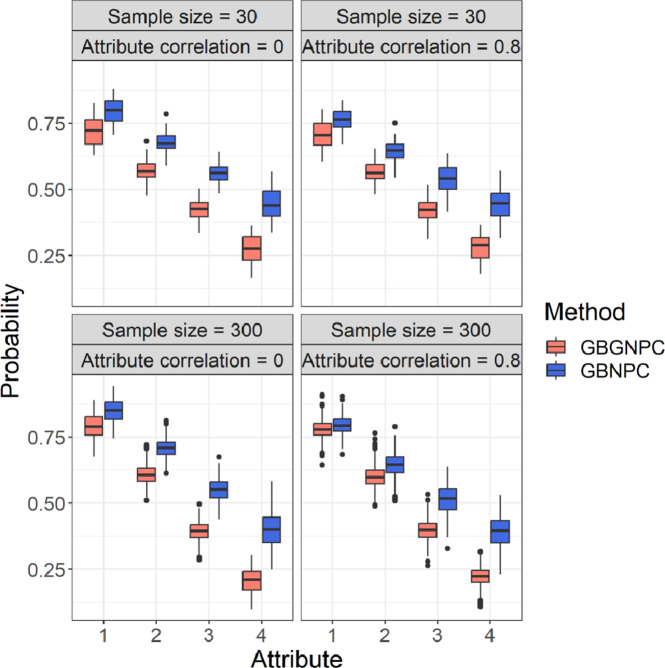
Box plots of attribute mastery probabilities of the general data generation with four-attribute 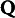 -matrix conditions.

**Figure 8:**
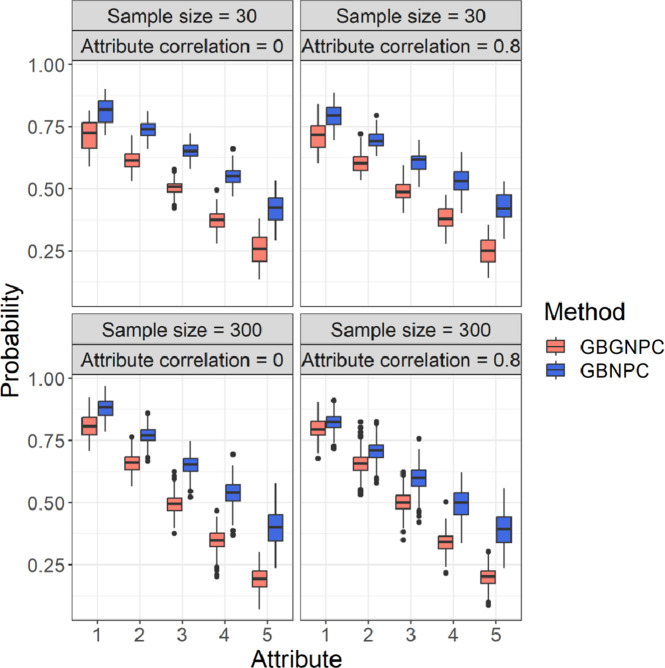
Box plots of attribute mastery probabilities of the general data generation with five-attribute Q-matrix conditions.

We also checked attribute mastery probabilities of the GBGNPC and GBNPC methods that represented uncertainty of parameter estimates. Figures 5 and 6 represent box plots of average attribute mastery probabilities of the four- and five-attribute conditions under the DINA model-based data-generating process. Interestingly, the GBNPC method tended to show higher average attribute mastery probabilities than the GBGNPC. The differences between the GBGNPC and GBNPC methods were relatively small in the first attribute but the discrepancy became larger as the attribute number increased. The later attributes were more difficult to master and the number of individuals mastering them was small. These tendencies also occurred in the general data-generating process situations, which are shown in Figures 7 and 8. These posterior probabilities of attribute mastering represent estimation uncertainty, so we can carefully check the attribute mastery status. For example, the attribute mastery probabilities around cutoff values might represent indeterminacy of mastery or nonmastery. Such uncertainty quantification results cannot be obtained through the GNPC or NPC methods.

In summary, NPC and GBNPC tended to have higher AARs and PARs under DINA data generation, low-item-quality conditions, and high-attribute correlations. However, GBGNPC was sometimes similar to NPC under the DINA data generation conditions, whereas GNPC was the least optimal. By contrast, under general DCM data generation conditions, GBGNPC and GNPC performed better than GBNPC and GNPC for high-quality items. For low-quality items, GBGNPC and GBNPC performed better. Based on these results, GBGNPC appears the optimal choice for attribute mastery estimation. If the DINA type item response mechanism is confirmed, GBNPC is the optimal choice among the four estimation methods from the perspective of attribute recovery.

The possible reason for the superiority of the GBGNPC over the GNPC is prior settings. In our simulation setting, sample sizes were relatively small in the situations in which the nonparametric methods were employed. Under such conditions, estimation of weight parameters might be difficult for the GNPC, especially under the low-item-quality conditions. The GBGNPC, on the other hand, assumed priors for the weight parameters, and the prior conveyed information of item characteristics and succeeded in estimating attribute mastery patterns. Another reason may be that the GBGNPC can deal with uncertainty in parameter estimation. This means that the GNPC uses parameter estimates to minimize the loss function, which simply selects the attribute mastery pattern that provides the minimum value of loss function without considering the second or third best attribute mastery patterns. By contrast, the GBGNPC can consider and use the second-best attribute mastery pattern for estimating attribute mastery probabilities. If these considerations are correct, even if we use noninformation priors for the GNPC method, the GBGNPC may remain superior. The effects of prior settings are also an important topic for detailed research in future studies.

In addition, the effects of manipulated factors are discussed here. First, attribute correlation affected attribute mastery recovery. The GB method provided better attribute mastery recovery than the nonparametric methods. The nonparametric loss could not include such information, but the generalized posteriors have such information based on the data. The attributes generally correlate with each other, making the GB methods generally better than the nonparametric methods. We did not explicitly include a loss related to the attribute correlations, but the GB method allows us to include a loss term of the attribute correlations or attribute structure. This may be a good extension for constructing a loss function for the GB method.

Second, item quality also affected attribute mastery recovery results. The GB methods showed better results than the nonparametric methods, especially under low-item-quality conditions. Under such conditions, prior information might help to improve attribute recovery. This means the GB method can utilize not only the loss function but also prior information. This makes the GB method the preferred method compared to the current nonparametric methods, which cannot do this. Thus, based on the simulation study, the GB methods are always better than the nonparametric methods from the perspective of attribute mastery recovery.

## 6 Real data example

The real data example aimed to compare the four estimation methods used in the simulation study and examine how these estimation methods provide different attribute mastery results. This real data comparison provided an example of the behavior of the proposed GB method for DCMs.

To show the superiority of their proposed methods, Ma and Jiang (2021) used 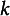 -fold cross-validation with the log marginal likelihood. From our understanding, the log marginal likelihood does not contain individual parameters that are attribute mastery patterns. In the cross-validation procedure, model parameters estimated with a training dataset are plugged in to calculate the log marginal likelihood of the test dataset. In our context, the loss functions in the GB method and nonparametric methods do not contain model parameters and only estimate attribute mastery patterns that relate to individuals. Therefore, the attribute mastery patterns in a training data set are not contained in a test dataset. Exploring the appropriate quantitative evaluation for the GB method in the DCMs is an important direction for future research.

### 6.1 Data analysis settings

The Examination of the Certificate of Proficiency in English (ECPE) data were selected as an example. ECPE data have been analyzed in various previous studies, such as Templin and Hoffman (2013) and Templin and Bradshaw (2014). The ECPE data contained 2,922 responses for 28 items. Table 4 presents a 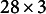 Q-matrix that assumes three attributes: Morphosyntactic 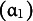 , cohesive 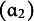 , and lexical rules 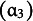 . The settings of the GB methods were the same as those used in previous simulations. One difference was that we employed GNPC and NPC estimates as initial values for GBGNPC and GBNPC. The data analysis code can be obtained from the OSF webpage https://osf.io/sau6j/.

**Table 4:** The 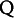 -matrix of ECPE data

Item	Attribute			Item	Attribute		
		Morphosyntactic	Cohesive		Lexical		Morphosyntactic	Cohesive	Lexical
	rules: 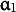	rules: 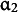	rules: 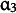		rules: 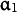	rules: 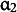	rules: 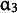
1	1	1	0	15	0	0	1
2	0	1	0	16	1	0	1
3	1	0	1	17	0	1	1
4	0	0	1	18	0	0	1
5	0	0	1	19	0	0	1
6	0	0	1	20	1	0	1
7	1	0	1	21	1	0	1
8	0	1	0	22	0	0	1
9	0	0	1	23	0	1	0
10	1	0	0	24	0	1	0
11	1	0	1	25	1	0	0
12	1	0	1	26	0	0	1
13	1	0	0	27	1	0	0
14	1	0	0	28	0	0	1

### 6.2 Results

The same correlations as in the simulation study were calculated. Again, the correlations of the three attributes with the GBNPC and GBGNPC methods were all greater than 0.99. This indicated that the MCMC iterations for attribute mastery were stable.

Table 6 lists the frequencies and ratios of the attribute mastery patterns for the four estimation methods. Several differences are observed in Table 6. First, GBGNPC and GBNPC estimated the pattern (001) to be lower than GNPC and NPC estimates. Second, as pattern (011) indicates, the frequency of pattern (011) for GBGNPC was the highest (1203), that for the GNPC was the second (955), that for the NPC was the third (522), and that for the GBNPC was the last (386). The GBGNPC and GBNPC produced lower frequencies than the GNPC and NPC for patterns (100), (101), and (110). The final difference is indicated in pattern (111). GBGNPC and GNPC had relatively smaller numbers than GBNPC and NPC.

Table 5 shows the means and *SD*s of the attribute mastery probabilities for the GBGNPC and GBNPC methods. The attribute mastery probability for the first attribute (Morphosyntactic rules) of GBGNPC was mean 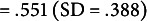 and that of GBNPC was mean 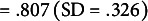 . The discrepancy was the largest among the three attributes. The attribute mastery probabilities for the second (cohesive rules) and third (lexical rules) attributes using the GBGNPC and GBNPC methods were higher than 0.90 so these attributes tended to be mastered.

**Table 5:** Means and SDs of posterior attribute mastery probabilities for GBGNPC and GBNPC methods

Estimation method	Attribute	Mean	SD
GBGNPC	Morphosyntactic rules: 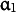	.551	.388
		Cohesive rules: 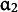	.985	.077
		Lexical rules: 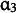	.939	.198
GBNPC	Morphosyntactic rules: 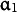	.807	.326
		Cohesive rules: 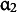	.978	.103
		Lexical rules: 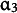	.949	.187

**Table 6:** Frequencies and ratios of the estimated attribute mastery patterns with the four estimation methods

Pattern	GBGNPC		GBNPC		GNPC		NPC	
		Frequency	Ratio	Frequency	Ratio	Frequency	Ratio	Frequency	Ratio
000	24	.008	35	.012	29	.010	44	.015
001	2	.001	3	.001	155	.053	91	.031
010	88	.030	64	.022	88	.030	82	.028
011	1201	.411	384	.131	955	.327	522	.179
100	3	.001	3	.001	38	.013	27	.009
101	0	.000	0	.000	82	.028	87	.030
110	45	.015	36	.012	157	.054	96	.033
111	1559	.534	2397	.820	1418	.485	1973	.675

*Note*: GBGNPC, generalized Bayesian method with generalized nonparametric loss function; GB-NPC, generalized Bayesian method with nonparametric loss function; GNPC, generalized nonparametric method; NPC, nonparametric method.

Table 7 shows the estimated attribute mastery patterns of GBGNPC and GNPC. A large portion of the GBGNPC pattern (011) corresponds to patterns (001), (001), and (010) of the GNPC. Furthermore, patterns (011), (100), (101), and (110) of the GNPC correspond to pattern (111) of the GBGNPC. From these results, the GBGNPC tended to overestimate the number of attributes compared with the GNPC.

**Table 7:** Contingency table of the estimated attribute mastery patterns by GBGNPC and GNPC

GBGNPC	GNPC							
		000	001	010	011	100	101	110	111
000	18	1	0	0	5	0	0	0
001	0	1	0	0	1	0	0	0
010	7	1	54	0	9	0	17	0
011	4	152	34	924	11	6	41	29
100	0	0	0	0	3	0	0	0
101	0	0	0	0	0	0	0	0
110	0	0	0	0	5	0	40	0
111	0	0	0	31	4	76	59	1389

*Note*: GBGNPC, generalized Bayesian method with generalized nonparametric loss function; GNPC, generalized nonparametric method.

Table 8 presents the GBNPC’s and NPC’s estimated attribute mastery patterns. The results in Table 8 are similar to those of GBGNPC and GNPC. For example, patterns (000), (001), (010), and (010) with the NPC are sometimes estimated as pattern (011) in GBGNPC. Furthermore, patterns (000) to (110) in the NPC were classified as pattern (111) in the GBGNPC. Therefore, the GBNPC overestimates the number of attributes compared with the NPC.

**Table 8:** Contingency table of the estimated attribute mastery patterns by GBNPC and NPC

GBNPC	NPC							
		000	001	010	011	100	101	110	111
000	26	5	1	0	3	0	0	0
001	0	3	0	0	0	0	0	0
010	10	0	37	0	8	0	9	0
011	7	54	32	278	3	0	10	0
100	0	0	0	0	3	0	0	0
101	0	0	0	0	0	0	0	0
110	0	0	2	0	3	0	31	0
111	1	29	10	244	7	87	46	1973

*Note:* GBNPC, generalized Bayesian method with nonparametric loss function; NPC, nonparametric method.

We checked individual differences between the GBGNPC and GNPC methods. Table 9 shows that some individuals indicated the largest pattern discrepancy of attribute mastery between GBGNPC and GNPC methods. The GBGNPC and the GNPC provided 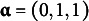 and 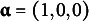 , respectively. The response patterns did not indicate systematic tendency but the sum scores of the individuals ranged from 11 to 15, which meant they could answer more than half of the test items. The maximum subscores for attributes one, two, and three were 13, 6, and 18, respectively, so the individuals in Table 9 received half points out of the maximum total subscores. In addition, the sum scores of the individuals ranged from 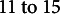 , which is about half the maximum sum-score of 28. Thus, the pattern 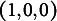 might saliently underestimate the latent attributes, making the pattern 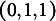 possibly more likely. Furthermore, some attribute mastery probabilities were close to the cutoff value 0.5. For example, the mastery probability of the third attribute for the ID 813 individual was 0.516. Additionally, the mastery probabilities of the first attribute for the ID 1060 and 2378 individuals were 0.418 and 0.420. Furthermore, the attribute mastery probability of the third attribute for the ID 2607 individual was 0.556. These values might indicate the mastery of corresponding attributes was not strongly supported. The posterior probabilities for the proposed GBGNPC and GBNPC methods can be used in such cases. However, this is not possible if we use typical nonparametric methods.

**Table 9:** Individual differences in estimated patterns for GBGNPC and GNPC methods, response patterns, sum- and subscores, and attribute mastery probabilities

ID	Attribute mastery		Response pattern	Sum-	Subscore			Attribute mastery		
		pattern				score				probability		
		GBGNPC	GNPC					α_1_	α_2_	α_3_	α_1_	α_2_	α_3_
813	011	100	1000100110100100000011101100	11	5	3	6	.130	.882	.516
1060	011	100	1000011101011000111010001101	14	7	3	9	.418	.956	.864
2378	011	100	1110110111110010010000001011	15	7	3	9	.420	.996	.982
2607	011	100	1000110000101000110010101100	11	5	3	7	.110	.874	.556

*Note*: GBGNPC, generalized Bayesian method with a generalized nonparametric loss function; GBNPC, generalized Bayesian method with a nonparametric loss function.

The attribute mastery probabilities information provides estimation uncertainty of mastery and nonmastery of an attribute for an individual. It may be better to empathize even if we judge an individual to have mastered an attribute as the mastery might be just slightly over the cutoff value. The nonparametric methods cannot provide such information. Therefore, it may be better to introduce the third category representing the midpoint between mastery and nonmastery in DCM applications.

In addition to each attribute mastery probability, we also added posterior attribute mastery pattern probabilities with the GBGNPC and GBNPC methods in Table 10. The individual’s posterior attribute pattern probabilities represent the relative possibilities of attribute mastery patterns. From Table 10, we can see that some attribute patterns showed almost the same posterior probabilities. For example, individual ID 2378 indicated relatively high posterior probabilities 0.574 and 0.406 for (011) and (111) according to the GBGNPC method. A similar tendency was shown by the ID 1060 students with the GBGNPC method. The posterior based on the GBNPC method provided more nuanced estimates for the ID 813 student. This individual had similar posterior probabilities for (000), (010), and (110), with values of 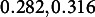 , and 0.250, respectively. It may not be better to provide diagnostic feedback with such unstable posterior probabilities. Previous nonparametric methods cannot provide such uncertainty information, which can be used for careful diagnosis of attribute mastery.

**Table 10:** Generalized posterior of attribute mastery pattern by GBNPC and NPC

Estimation method	ID	Attribute mastery pattern							
				000	100	010	110	001	101	011	111
GBGNPC	813	.102	.016	**.264**	.102	0	0	**.504**	.012
		1060	.008	.022	.020	.086	.006	.008	**.548**	**.302**
		2378	.002	0	.004	.012	0	.002	**.574**	**.406**
		2607	.104	.012	**.246**	.082	.010	0	**.530**	.016
GBNPC	813	**.282**	.012	**.316**	**.250**	.006	0	.106	.028
		1060	.022	.008	.004	.016	0	.004	.066	**.880**
		2378	.006	.002	.004	.016	.002	.002	.072	**.896**
		2607	**.520**	.028	.082	.054	.022	0	**.244**	.050

*Note*: GBGNPC: generalized Bayesian method with a generalized nonparametric loss function, GBNPC: generalized Bayesian method with a nonparametric loss function.

## 7 Discussions and future directions

This study extends the loss function-based estimation method proposed by Ma, de la Torre, and Xu (2023) to the GB method, which considers estimation uncertainty and prior knowledge. The proposed estimation method can be used for any type of loss function and has great flexibility. This study’s contribution is that the proposed method provides a novel approach for estimating the DCMs’ parameters. The GB method is flexible because we can select any type of loss function and consider the uncertainty of the parameter estimation. Furthermore, the proposed method relaxes the assumption of the typical Bayesian method, which requires a likelihood function. The theoretical analysis revealed consistent results for the proposed GB method under mild regularity conditions. Additionally, the simulation study revealed that the GB method improved attribute mastery recoveries compared to previous nonparametric methods. The real data example indicated that the proposed GB method with the nonparametric loss function tended to overestimate attribute mastery compared to the nonparametric methods.

The theoretical results not only guarantee the consistency of the MAP estimation results but also give convergence rate results, which is helpful in characterizing the finite sample estimate errors. All these results are new to the literature and provide theoretical justification for using the nonparametric methods and the proposed GB approach. Moreover, the theoretical results in the paper are established for the general loss function under the proposed assumptions. It covers popular loss functions, such as the GNPC and log-likelihood loss functions, which are used in Ma, de la Torre, and Xu (2023).

One interesting future research problem is to establish consistent results for other Bayesian estimators, such as expected a posteriori (EAP). However, this is a more challenging question as it involves deriving the limiting distribution of the Bayesian posterior distribution. Intuitively, given our theoretical results of MAP, EAP would also be consistent, but technically this is not easy to determine and needs the development of new mathematical tools. Moreover, Assumption 2 may be further relaxed to allow for some latent attribute mastery patterns that do not exist in the population. In particular, if we know which attribute mastery patterns have zero probability, such as in hierarchical DCMs, then our theoretical results would still apply. However, if this information is unknown, while some latent attribute mastery patterns have zero probability, the model itself may have some identifiability issues under the nonparametric DCM setting. This is another interesting topic for future study.

Another future research direction is to explore how to determine the learning rate from data, especially under the 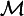 -open setting. Intuitively, the learning rate controls the relative importance between prior information and the loss function. We can set a relatively small value for the learning rate if we have enough prior information about the attribute mastery distribution and use several new items whose nature we do not know. In this case, we put relatively great importance on the prior information rather than the obtained data. However, it may not be realistic to set the learning rate greater than one. Such a high learning rate would amplify the effect of the loss function but might indicate an overreliance on the data. It may not be suitable for the 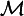 -open setting that the data-generating process is unknown. Therefore, we need to explore how to determine the learning rate from data.

As mentioned previously, no scholarly agreement exists regarding how to determine the learning rate, which is an important topic for future research especially in the DCM context. In particular, data-driven learning rate determination procedures were studied in Wu and Martin (2023), where several selection methods such as the SafeBayes algorithm based on the cumulative log-loss (Grünwald & van Ommen, 2017), information gain perspective (Holmes & Walker, 2017), modified weighted likelihood bootstrap approach (Lyddon et al., 2019), and the approximate achievement of nominal frequentist coverage probability (Syring & Martin, 2019) were compared. However, all of these methods have different foundations, and we need to explore which one is most appropriate for the DCM context.

Another topic that requires further investigation is model data fit evaluation. From our understanding, the GB method avoids explicit model representation in the framework. Therefore, the model evaluation scheme is not included in the procedure of the GB method. This is also true for the GBGNPC method proposed in this study. Therefore, future research needs to explore what kind of statistics can be used for model data fit. In particular, previously developed methods of model data fit assessment in psychometrics and Bayesian data analysis could be employed in our setting. Following Sinharay (2006), discrepancy measures such as observed score distribution, point biserial correlation, and statistical measures of association among the item pairs could be used for posterior predictive model checking (PPMC). For further details on PPMC methods for Bayesian networks and IRT models, see also Sinharay (2006) and Sinharay (2016). Moreover, PPMC for person fit (Sinharay, 2015) would also provide an important measure to assess the model fit for the attribute mastery patterns at the personal level, which is often of interest in cognitive diagnosis.

As a final note about the choice of estimation methods, it is necessary to consider estimation time. The GB method employs an MCMC procedure, so it has a longer estimation time than that of the nonparametric methods. In our simulation, the estimation times were less than ten seconds, so it is not irritatingly time consuming. However, if we need immediate feedback, the time difference between the two kinds of methods may be crucial. We also need to consider estimation time for the requirement of real data analysis.

## Data Availability

The data analysis code is available in the Open Science Framework page: https://osf.io/sau6j/.

## 1 Appendix

### A.1 Preparation for the Proofs

In this appendix, we provide some basic tools and introduce helpful notations for the proofs of Theorems 1 and 2. The proofs are presented in the subsequent sections.

Motivated by the constraint (34), we introduce the concept of a “local” latent class at the item level. Considering item  with *q*-vector  , the constraint (34) divides the collection of attribute mastery profiles  , which is  , based on an equivalence relationship where  is defined by  ; here, the subscript  emphasizes that the equivalence relationship is determined by the  th item  . On this basis, we introduce a function  where  is equivalent to  . This function assigns numbers to the equivalent classes induced by item  based on specific rules. In the following context, we refer to  as the local latent class of  induced by item  . It is straightforward to verify that the number of local latent classes induced by item  , denoted by  , is equal to  . Here,  represents the number of latent attributes required for item  ; consequently, the range of function  satisfies  . As the local latent classes are identified up to permutations on  , owing to their categorical nature, the mapping rules between  and  need not be completely specified in our discussion.

For brevity, we use the general notation  to denote the collection of local latent classes for all items  and subjects  , where  represents  . Given that  implies  by the definition of  , we express  as  to directly incorporate the constraint (34) into the loss function (31). For notational simplicity, we may write  as  . Consequently, we define:

(A.1)

Then, the loss function (31) can be rewritten as:

(A.2)

where  . Observe that  , and  , we denote the expectation of the above  by  .

Notably,  is determined only by  because  is known. In the subsequent context, the quantities determined by the latent attribute profiles  are sometimes denoted by the superscript  to emphasize their relationships with  . Considering an arbitrary  , we denote it as:

(A.3)

(A.4)

where  and the definition of  is provided later. Notably,  may not minimize  for a given  . Then, under any realization of  , if the prior distribution of  is uniform, the following equations hold for any local latent class  :

(A.5)

To derive (A.5), note the sum  equals the sum  . When estimating  , we focus on minimizing  . By substituting  into (A.5), we find that  holds for any  . In the following section, we use the second formula in (A.5) to define  given in (A.4). When the prior distribution is not a uniform distribution,  is obtained from minimizing  , where  is the prior density of  . To avoid ambiguity, we denote  , and  . It is clear that  .

Before discussing the details of our proof, we provide technical remarks to simplify the discussion. Notably, although we assume that the latent attributes have proportion parameters  , they are still treated as unknown but fixed parameters that need to be estimated. As all the proportion parameters  are strictly greater than zero, with the probability converging to  exists such that  . Subsequently, we use this fact interchangeably with the first condition in Assumption 2.

The second point concerns the compact parameter space specified in Assumptions 2 and 3. Some loss functions may exhibit unusual behavior near the boundary of the parameter space. Although Assumption 2 confines the true item parameters to a compact subset within  , the estimated item responses can still approach zero or one, making theoretical analysis more difficult. For any pair  lies within  . We add a condition to  , stating that there exists an  such that for each  , the sum  is at least  . With a probability approaching one, this constraint is satisfied by the true latent attribute mastery patterns  . With this constraint, for any pair  , the probability that  exceeds  can be bounded by  using Hoeffding’s inequality. Thus, the probability that  exceeds  is less than  . Based on the scaling condition in Theorem 1,  , implying that with probability converging to 1, all the  values fall within  . Based on this result, we assume that in the later content, the estimators  are obtained by minimizing the total loss (A.2), under the constraints that  and  , for two small positive constants  .

The third comment concerns how to quantify the effect of prior density  on the corresponding estimator  . Actually, under the smoothness and shape constraints given in Assumption 5 and Assumption 3, the additional term  might cause the estimator  to have a  level drift from the sample average form  given in (A.6). By considering the Taylor expansion formula, we have:

where  is between  and  according to the mean value theorem; the second equality holds owing to Assumption 3. According to the above equation, we can find that  . Note that if we take  . Based on Assumption 5, there exists a constant  such that  , implying the following:

where  . Thus, a constant  exists such that for any pair  , we have:

This inequality will be used several times afterwards. In the theoretical analysis of the estimators above, uniform bounds related to the quantities of element-wise loss  and prior densities  are frequently used. The existence of these uniform bounds requires restricting the parameter space of the item response parameters to a compact subspace. Therefore, discussing the compact parameter subspace of the item parameters is necessary.

### A.2 Outline of the first half of the proof

Step 1: Express the upper bound of  in terms of  , where  depending on  and  under  is the upper bound of the second-order derivative of the  .
Step 2: Bound  and  separately to obtain a uniform convergence rate

Step 3: Based on the definition of  , it follows that  , which controls the deviation  .

In some classical statistical inference contexts, consistent results for the parameters of interest are typically established through the uniform convergence of random functions associated with these parameters. For instance, if  , and if we further assume that  has a unique minimum  on  under some regularity conditions. The regular conditions may vary across settings. Considering  as the parameter to be estimated, the primary aim of the first three steps is to demonstrate that  minimizes the expected loss and establishes a uniform convergence result for its random loss function of  .

### A.3 Outline of the second half of the proof

Step 4: Define  to represent the samples with the wrong local latent class assignments. Derive some upper bounds for the quantities based on  using  with the help of the identification assumptions.
Step 5: Bound the  using the quantities based on  with the help of the discrete structure of the Q-matrix, then obtain the desired classification error rate.

Assumptions 1–5 are the regularity conditions for achieving clustering consistency based on the uniform convergence results established in the first half of the proof. We have provided further details regarding the assumptions in later proofs.

### A.4 First Half of the Proof of Theorem 1

**Step 1.** The idea of decomposing  is to consider:

The variability in the first term of the right-hand side mainly emerges from the fluctuation in  , while the randomness in the second term is attributable to the stochastic nature of  . Lemma 1. Let  denote independent Bernoulli trials with parameters  . In a general latent class model, given arbitrary latent attribute mastery patterns  ,

(A.6)

where  *is a random variable depending on*  *and*  *denotes the number of the distinct local latent classes induced by*  *for item*  . Proof. By noting the decomposition that we mentioned at the beginning of Step 1,  is easy to check. It is sufficient for us to prove that

The first inequality is clear by the definition of  (minimizing the loss). For the second part, using the mean value theorem for second-order derivatives, we obtain

(A.7)

where  is between  and  according to the mean value theorem. The third equality holds true since by Assumption 3, we have

Similarly, using  , we have:

which concludes the proof of this lemma. 
Lemma 2. The following event happens with a probability of at least  ,

Proof. Under any realization of  , each  is an average of  independent Bernoulli random variables  with mean  . By applying the Hoeffding inequality, we have

(A.8)

Notably, considering a fixed  , each  can take values only in the finite set  of cardinality  . We denote this range of  by  and the range of the matrix  by  . Subsequently,  for any  . As each of the  entries in  ,  can independently take on  different values, there is  with constraint  . As  , we have  . Denote  , and

(A.9)

The above result holds for any fixed  when we apply a union bound over all the  possible assignments of  to obtain

(A.10)

Take  ; then,  . This concludes the proof of lemma 2. 
Lemma 3. Define the random variable  , and denote  . Note that  and  are continuous on  in  . Since continuous functions on the compact set are bounded, a constant  exists such that  . By applying Hoeffding’s inequality to bound  for any realization of  , we have:

(A.11)

With the help of Lemma 2 and Lemma 3, subsequently, we prove the following proposition: Proposition 1. Under the following scaling for some small positive constant  ,

we have  where  for a small positive  . Proof. First, note that under the given scaling condition,  . Combining the results of Lemma 2 and Lemma 3, since there are  possible assignments of  , we apply the union bound to obtain

(A.12)

For the third term, note that the following inequality holds for any given  :

For the second term on the right-hand side of the aforementioned display to converge to zero, we set  for a small positive constant  . Moreover, under this  , for the first term to converge to zero as  increase, the scaling  given in the theorem results in  , which implies the result in Proposition 1.

Step 3. (A.5) implies that  , which means that if we plug into the true latent attribute mastery pattern  , the estimators will be the corresponding true parameters. According to this property, the following lemma indicates that  minimizes expected loss.
Lemma 4. By Assumption 4,  for some  , then we have

(A.13)

Notably, while Lemma 4 holds for any  , it also holds for the estimator  , then

(A.14)

As  , we have  . Substituting this into A.14, we can derive that

### A.5 Second Half of the Proof of Theorem 1

By applying Hölder’s inequality, we have

By letting  , we can check that  where  . In the following, we derive a lower bound for  because it is easier to work with than  .

**Step 4.** Motivated by Assumption 2, we define  s.t.  , which represents the set of all items  that depend on only one latent attribute. Notably,  , as  only contains one required latent attribute, then  for all  . Without loss of generality, we assume that if  , then let  , otherwise, let  . Further, we assume that  , which aligns with the concept that subjects possessing the required latent attribute tend to perform better. For any  , define

(A.15)

Note  and  ,  . By using (A.5), there are

(A.16)

Under  , we impose a natural constraint  on  for identifiability purpose. This constraint does not change the previous results as  allows  in (A.14) still holds; thus,  still holds under this constraint. Combining  and  , there is

(A.17)

From (A.15), we can obtain

Therefore,

(A.18)

The second inequality holds since by Assumption 1,  . One ideal scenario is that for most  ; thus the misclassification error for the local latent classes could be bounded relatively tight. The following result confirms this intuition. Lemma 5. Define the following random set depending on the estimated latent attribute mastery patterns  under constraint  :

then under Assumption 1 and Assumption 2, there are  .

**Proof.** If  . Under Assumption 2,

then

By noting  , then  . Similar arguments yield  , which concludes the proof of Lemma 5.

Note (A.17) implies that  , thus  . Lemma 5 implies that when  goes to 0 with a mild rate, the number of elements in  dominates the number of elements in  ; thus for most  should be  , which represents the number of subjects with the incorrectly assigned local latent classes. Step 5. (A.18) implies that  . Next we focus on obtaining a lower bound of  to control the classification error rate  .

Motivated by Assumption 2, for each latent attribute  , denote  as the smallest integer  such that item  has a  -vector  , and denote  as the second smallest integer  such that  , etc. For positive integer  , denote

(A.19)

For each  , denote

(A.20)

Then, we have that  for any  , thus

(A.21)

The last inequality holds since  . Note (A.21) implies  . For simplicity,

Note that (32) in Assumption 2 implies that  and  by plugging these results into  , we can obtain

From Lemma 5, we have  for  , which implies that  . By substituting this into the above inequality, we can conclude that

which is equivalent to  . The proof of this theorem is complete.

The inequality (32) in Assumption 2 bridges between the misclassification error for the local latent classes and the misclassification error for the latent attribute mastery patterns  by using the inequality  .

### A.6 Proof of Theorem 2

For notational simplicity, denote  . Thus, Assumption 2 implies that  and

(A.22)

Recall that

Rewrite  as similar form

By triangle inequality, we have

Thereafter, we analyze these three terms separately. For the first term,

The last inequality holds since  . For the second term, we have

For the same reason as  , we can also conclude that  ; thus,  . For the third term, we apply Hoeffding’s inequality for bounded random variables and obtain

Note the number of  pairs less than or equal to  under Assumption 2, we have for  ,

(A.23)

Notably,  remains a constant as  is fixed. By choosing  for a small  , the tail probability in A.23 converges to zero when the scaling condition  holds. This implies that  . Bringing together the preceding results, we have

Note for any  , we have  and  with the probability approaching 1 ; thus  . Therefore,
